# A low-cost, high-performance method for the production of particles to trace water flow with high contrast in XROMM studies

**DOI:** 10.1242/jeb.252187

**Published:** 2026-07-13

**Authors:** Lukas Hageneder, Andres Vanhooydonck, Regan Watts, Sam Van Wassenbergh

**Affiliations:** ^1^ University of Antwerp, Department of Biology, Functional Morphology, 2610 Antwerp, Belgium; ^2^ University of Antwerp, Department of Product Development, 2000 Antwerp, Belgium

**Keywords:** Fluid dynamics, Functional morphology, Flow tracers, XROMM, X-ray attenuation, X-ray particle tracking velocimetry, XPTV

## Abstract

Dynamic X-ray imaging has proved to be a useful method for tracing particles in otherwise obscured areas. Through the combination of X-ray Reconstruction of Moving Morphology (XROMM) with X-ray Particle Tracing Velocimetry (XPTV), new insights in functional morphology can be gained. Creating particles with desired properties as neutrally buoyant but yet different from water in their attenuation of X-rays has proved to be a crucial but challenging task. This paper describes a simple and low-cost protocol for the manufacture of XPTV particles of specific diameters to fit the application. This is achieved through a novel approach where the particles are made from a homogeneous mixture, allowing them to be shaped as needed, in contrast to existing solutions that rely on assemblies of at least two precisely dimensioned components. The proposed approach satisfies key characteristics, including high X-ray attenuation, uniform shape and specified effective density, and outperforms current state-of-the-art solutions.

## Teaser

**Summary:** A novel single-mixture protocol for manufacturing neutrally buoyant X-ray tracer particles of specified diameter, offering a simpler and more versatile alternative to existing approaches.

## INTRODUCTION

The advantages of X-ray Particle Tracing Velocimetry (XPTV) compared with classical Particle Image Velocimetry (PIV) or Particle Tracing Velocimetry (PTV) have attracted increasing attention in recent years. As reviewed by Drake et al. (2011), there are many different applications for XPTV, from studying the flow and mixture in fluidized beds (Morgan and Heindel, 2007, 2010), to analysing chemical processes in packed beds (Owens et al., 2009) and slurry beds (Shimada et al., 2007), as well as performing high-resolution tracing using monochromatic X-ray sources (Lee and Kim, 2003; Im et al., 2007). This has been accompanied by the need for suitable tracing particles that can reliably and automatically track fluid flow. Opaque fluids are commonly found in various places, from foams, to fluids constrained by the containers in which they flow, via non-transparent liquids or liquid–gas mixtures (Drake et al., 2011; Kim and Lee, 2004; Lee and Kim, 2003; Bruni et al., 2002). Generally, all these different approaches for creating tracing particles aim to achieve the following properties: (1) different in X-ray attenuation from the surrounding medium; (2) the same mass-density/properties as the surrounding medium; (3) small enough to capture the scale of flow patterns of interest

Properties 1 and 2 are difficult to combine, as materials with higher X-ray attenuation are typically heavier, and vice versa for those with lower attenuation. Also, 1 and 3 are in trade-off as larger particles will logically attenuate more.

Various types and sizes of particles have been designed and used in engineering studies. Drake et al. (2011) and Chen et al. (2019) investigated fluidized beds, which are solid particles suspended by upward gas flow, creating fluid-like behaviour. Drake et al. (2011) followed a neutrally buoyant particle, which they created by surrounding a 2 mm lead ball with expandable foam to a total size of 8–9 mm, whereas Chen et al. (2019) followed particles by putting a metal rod in the centre of the particle. Kingston et al. (2014, 2015) investigated the mixing of wood chips in a double screw mixer, where they stained chips with potassium iodine and coated them with silver for more contrast in the X-ray. On a smaller scale, Mäkiharju et al. (2021), Parker et al. (2022) and Bultreys et al. (2022) used micro-computed tomography (CT) scanners in combination with tracing particles of around 50 μm in size. Mäkiharju et al. (2021) and Parker et al. (2022) used silver-coated micro-glass spheres for tracing, while Bultreys et al. (2022) developed hollow carbon spheres coated with tungsten, offering better contrast in X-ray images due to tungsten's higher attenuation compared with silver. This shows the wide variety of tracing particles with very specific and different requirements focused on the problem.

As none of the above particles were suitable for tracking water flow using bi-planar image intensifiers, Provini et al. (2022) developed a new type to be used to study suction feeding in fish. These particles needed to be neutrally buoyant, small enough to be ingested by the fish, and visible through several centimetres of water during fast movements, as the flow speed during suction feeding could exceed 1 m s^−1^. To achieve this, the particles were designed using EPS foam spheres (1.4 mm in diameter) punctured with a brass rod (0.4 mm diameter), resulting in a density of 994±33 kg m^−3^, which is close to that of water but with a broad distribution. Consequently, a large number of particles had to be produced to ensure enough were near neutral buoyancy. Another challenge stemmed from the particles' one-dimensional shape, as their cross-section changed significantly during rotation, complicating automatic tracking. The aim of the present study was to formulate a new production procedure and benchmark the particles developed by Provini et al. (2022), as these best meet the key requirements for an X-ray Reconstruction of Moving Morphology (XROMM) (Brainerd et al., 2010) setup, including appropriate size, high visibility during fast motion, and neutral buoyancy. The new production method seeks to reduce the time required for particle manufacturing, minimize variation in their mass-density and achieve a fully homogeneous density distribution, allowing for the tracking of a stable, non-changing point. By optimizing these factors, the study aimed to create more consistent and reliable particles for water flow tracking in high-speed environments, thereby enhancing the overall efficiency and accuracy of XROMM applications.

## MATERIALS AND METHODS

The new particles were produced, tested for their density and benchmarked for visibility and traceability in the high-speed X-ray videos that are used in XROMM.

### Production

The goal of producing new particles was to make the process more reliable and reduce the manufacturing time needed per particle. This was achieved by dividing the process into three steps: (1) the creation of the composite material, (2) the formation of the desired shape of the tracing particle and then (3) hardening of the material. This allows for fine control over the composition of the material and makes it easy to adapt for various situations.

The composite material consists of four materials: tungsten powder, microballoons, liquid clay and colouring. The tungsten powder has a high X-ray attenuation and creates the contrast needed to track the particles. The microballoons are N_2_ inflated micro-plastic spheres to counterbalance the weight of the tungsten. The liquid clay gives stability and connects the different parts of the material. Through the properties of the liquid clay, the material has a dough-like consistency and is very easy to form into the desired shape. Once it is shaped as desired, the material is put in an oven for hardening, giving the particles the needed rigidity. The fourth component, colouring, allows more versatile applications, as this might play a role when working with animals. The exact composition and the materials used are shown in Table 1 (see also Supplementary Materials and Methods, ‘Recipe’). The individual components are generally considered low or non-toxic materials. However, the biocompatibility of the final composite material has not been formally evaluated.

**Table 1.** Tracing particle components

Components	*d*_avg_ (μm)	Brand name	*w* (%)
Tungsten powder	12	Thermo Scientific: APS 12 micron 99.9% (metal basis)	71.936
Microballoons	93	Dualite: E 035-FR	3.578
Liquid clay	–	Fimo: liquid	18.398
Colouring	–	All Acryl Amsterdam: red	6.089

*d*_avg_ is the mean particle diameter of the spherical powder and *w* is the weight percentage with an uncertainty of Δ*w*=0.002%.

**Fig. 1.**
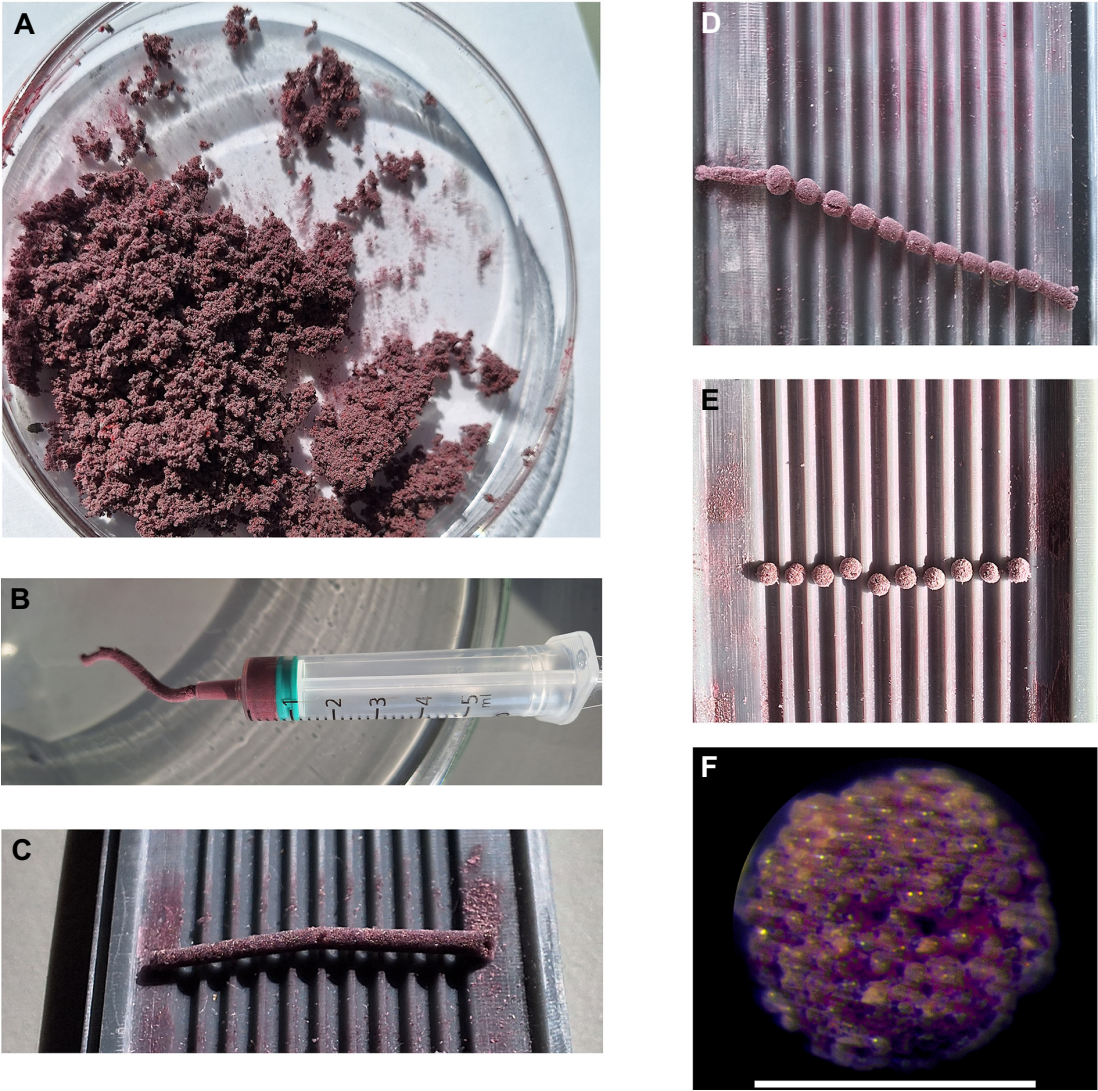
**Manufacturing process for tracing particles.** (A) The homogeneously mixed components of the composite material. (B) Extrusion of the composite material through a syringe. (C) The rolled out cylinder, which is then placed on the 3D printed model (D); through rolling back and forth, rills will be created (E). (F) The end product viewed under a microscope. Scale bar: 1 mm.

The components were mixed with a silicon spatula for 10 min; after this time, the mixture was visually homogeneously mixed (Fig. 1A). In this process, the amount of shear forces was kept as low as possible – in particular, we avoided pressing the spatula against the bottom of the container and dragging it across – as this has a negative impact on the density of the particles. Subsequently, the mixture was filled into a syringe with a Luer lock and an opening of 2 mm. The material was extracted through the opening of the syringe and thus compacted into the shape of a cylinder with a diameter of 2 mm (Fig. 1B). This cylinder was then placed on a glass pane, with two distance holders, one on each side. With a second glass pane, that was softly pressed against the extruded cylinder without flattening it, the diameter of the cylinder was gradually reduced by gently rolling it back and forth. The desired cylinder diameter depends on the desired particle size; ultimately it should be roughly 87% of the intended sphere diameter to be formed in the next step.

The formed cylindrical roll was placed on top of the mould, as shown in Fig. 1C, with additional images provided in Fig. S1. Importantly the roll was placed perpendicular to the rills on top of the mould. The protruding sections of the roll were trimmed with a scalpel at the boundary of the grooved region to prevent smearing of the mixture along the sides. The second part of the mould was then placed on top of the cylinder and again with gentle movements back and forth, the cylinder was slowly pressed into the rills of the mould, creating the spheres. This was done until there was no more space between the two moulds (Fig. 1D,E).

The spherical particles were then put into the oven at 130°C for 20 min as suggested by the manufacturer of the liquid clay to harden it. Subsequently, they were stored in water for 7 days to reach a state where the density reached an equilibrium close to the density of water after being fully saturated. The ready to use particles were stored in water, to maintain their waterlogged state. A picture of the finished particle under the microscope can be seen in Fig. 1F.

The production time and per-particle costs were drastically reduced, thereby enabling faster production at a lower cost. Provini et al. (2022) informed us that they were able to produce approximately 60 particles per hour, compared with around 150 particles per hour achieved with the new method. This time was measured for 1 mm particles; smaller particles generally require more production time as handling becomes increasingly difficult. Additionally, Provini et al.’s (2022) costs amounted to approximately €100 for tools and €200 for a precision sieve to filter the exact size of Styrofoam spheres. The material costs were around 1–1.4 € cents per particle. In comparison, the new method required around €30 for the oven and approximately €60 for a 3D-printed mould for a single size. The material costs used in the recipe amounted to €286 per 1 kg of mixture, with tungsten powder costing €187 per 0.5 kg, while microballoons were obtained as a free sample; furthermore, 120 ml of acrylic paint cost €7.35 and 200 ml of liquid clay cost €16.2. This resulted in a price per tracing particle of 0.179 € cents. In summary, the production time was reduced by more than half, while material costs allowed more than 5 times as many particles to be produced for the same price.

### Validation

To validate the developed production protocol, particles were produced and tested for density. This was repeated for four different particle sizes: 0.5, 1, 1.5 and 2 mm in diameter (*d*). For each size, the following number (*n_d_*) were produced: *n*_0*.*5_=39, *n*_1*.*0_=100, *n*_1*.*5_=100, *n*_2*.*0_=100. The particles were submerged in a 25 cm high water tank and were recorded on video. The footage was analysed with tracking software (XMALab version 2.1.0; Knörlein et al., 2016) and velocities were calculated. When the terminal velocity was reached, this value was used to calculate the mass-density of the particles (ρ_p_) with the Stokes equation (see Eqn 1). As the velocity $\vec{v}$ does not change any more, the acceleration is zero, and therefore the buoyancy *F*_b_ and the drag *F*_d_ cancel each other out: *F*_b_=*F*_d_. With the known density of the fluid ρ_f_ and the radius of the particles *r*, it is possible to calculate the density of the particles ρ_p_:

(1) $$\begin{array}{rl} & \;F_{b}=(\rho_{p}-\rho_{f})g\frac{4}{3}\pi r^{3},\\ & F_{d}=-6\pi \mu \;r\vec{v},\\ & F_{b}=F_{c}\Rightarrow \vec{v}=\frac{2}{9}\frac{\rho_{p}-\rho_{f}}{\mu }gr^{2}. \end{array}$$

To validate the traceability of particles under varying water depths, the system for XROMM at the University of Antwerp, featuring a high-speed X-ray imaging system, was employed. This system has been described in detail by Sanctorum et al. (2019). Its imaging method is representative for image-intensifier or C-arm-based XROMM systems. In brief, the system uses Philips SRM 0511 X-ray tubes with Philips XD6028 collimators and a Philips Imagica HC 38 cm diameter image intensifier. The image intensifier's output screen is filmed with a Photron FastCam Mini WX50-32 GB high-speed camera at 750 Hz at 2048×2048 pixels. The testing of the system showed a specific resolution of a maximum 2.5 line pairs per mm at the widest field of view. This XROMM system was used to validate the traceability of the tracing particles in a single detector plane under various water ‘depths’ (i.e. the distance through the water along the X-ray beam). This was done by putting a Styrofoam plate, which the particles were taped onto, on top of a horizontally mounted detector and accelerating it by dropping a heavy object that was attached to it. This setup allowed us to put a glass tank with 0.5 cm thick walls 4 cm above the plate and detector and fill it gradually with water for testing of various water depths. This controlled setup allowed precise evaluation of how water depth and speed influence particle traceability, ensuring reliable validation of the XROMM system under dynamic aquatic conditions.

The key property for tracing particles is their actual traceability, and we therefore analysed all datasets to assess this property. To avoid bias, the only image enhancement applied was standard flat-field correction. The corrected image sequences were then imported into XMALab – currently the state-of-the-art software for automated XROMM marker tracking. Marker detection was performed using the ‘default X-ray marker’ setting. Traceability was assessed using the following metric: if the software lost track of a marker more than twice within a span of five frames, tracking was considered unreliable beyond that point. The speed one frame before the second occurrence of a loss was then recorded as the highest speed at which it could be reliably tracked. To enable direct comparison, four different particle size categories were tested alongside the particles described in Provini et al. (2022). Three of these reference particles were mounted in different orientations on a Styrofoam plate, as their internal rod structure causes their X-ray appearance to vary with orientation. Together, these validation steps ensured that the particles could be reliably detected and tracked under varying experimental conditions, confirming their suitability for use in high-precision flow studies.

Differences between groups were assessed using the permuted Brunner–Munzel test with the user-contributed Brunner–Munzel-test-for-matlab repository on GitHub for MatLab. This is a non-parametric method that tests the stochastic equality of two distributions without assuming equal variance or equal distributional shapes (Brunner and Munzel, 2000; Neubert and Brunner, 2007). The permutation-based approach provides valid inference even for small sample sizes, where the asymptotic approximation of the original Brunner–Munzel test may be unreliable. This test was particularly suitable for our data, as group distributions differed in both shape and variance due to a ceiling effect at the maximum measurable velocity.

## RESULTS AND DISCUSSION

To examine the effect of particle size on density, four different particle sizes (0.5, 1.0, 1.5 and 2.0 mm) were tested by measuring their terminal velocity in a water column. Using the Stokes equation (see Eqn 1), the density of the particles was then calculated. The equation also reveals that the terminal velocity is directly proportional to the density, hence revealing a weak dependency of the mass-density on the particle size, with a noticeable difference between the 0.5 mm particles, with a density of 1180±60 kg m^−3^, and the larger ones; the 1.0 mm particles had a density of 990±40 kg m^−3^, the 1.5 mm ones had a density of 1000±20 kg m^−3^ and the 2.0 mm particles had a density of 992±10 kg m^−3^, indicating only a slight variation. The recipe for these particles was designed for 1.5 mm sizes, but it is important to note that the plastic spheres used to balance the tungsten powder in the smaller 0.5 mm particles are relatively large. In fact, only about 80 spheres, assuming random closed packing, are needed to fill a single particle. This means that even a single plastic sphere more or less influences the total density by approximately 1.25%. While particle size does influence the observed terminal velocity, the effect is most pronounced in the 0.5 mm particles. This highlights the importance of carefully considering particle composition and balance when adjusting the recipe for a different use case, including considering smaller microballoons. However, this comes at the price of a slightly higher density of the microballoons but a better homogeneity.

The study also accounts for the spatial heterogeneity due to asymmetry of state-of-the-art (SoA) particles with respect to the X-ray beam axis, which was examined across multiple orientations to assess its impact on the signal-to-noise ratio (SNR) (see Eqn 2). This describes the detectability, the second key quantity, which was calculated to compare the SoA particles described in Provini et al. (2022) and the newly developed particles. To quantify this, 12 measurements were analysed for every particle size and orientation in the case of the SoA particles. Through analysing a line spectrum through the centre of the particles, the SNR was computed (Shannon, 1948):

(2) $$\mathrm{SNR}=\frac{\mu_{s}^{2}}{\sigma_{n}^{2}}=\frac{P_{\mathrm{signal}}}{P_{\mathrm{noise}}}.$$

**Fig. 2.**
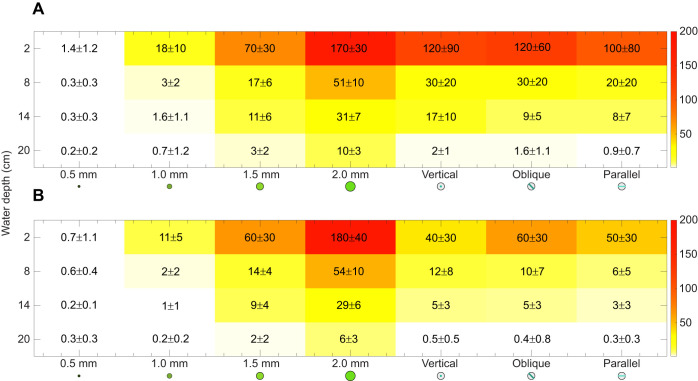
**SNR comparison of state-of-the-art (SoA) and new tracers across varying water heights.** Heat map for the SNR for the static case (0 m s^−1^; A) and the dynamic case (1 m s^−1^; B) for the four different particle sizes: 0.5, 1, 1.5 and 2 mm diameter. The SNR is given with an uncertainty ΔSNR, for multiple measurements around the estimated centre of the sphere.

Here, μ_s_ is the mean value of the signal peak, and σ_n_ is the root mean square of the noise taken from a signal-free region in the vicinity of the particles. These results emphasize that both particle size and homogeneity play a crucial role in optimizing SNR, thereby offering valuable guidelines for the design of next-generation particles with improved detection performance. The comparative analysis reveals clear differences in SNR between the tested particles, and between the static versus dynamic conditions (Fig. 2).

**Fig. 3.**
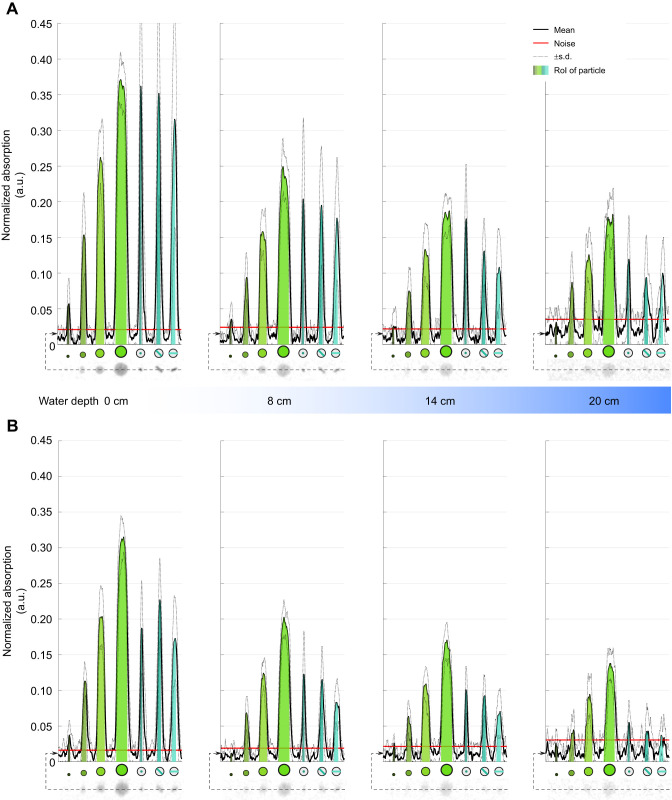
**Contrast assessment by comparison of absorption through the centre of a patch with all particles aligned.** An example X-ray image is shown below each absorption; the dashed line indicates the centre. The absorption (in arbitrary units, a.u.) was calculated through the grey value of the pixels and normalized to a range from 0, corresponding to the flat-field intensity (no absorption), to 1, corresponding to zero transmitted intensity (full absorption). (A) The static case (0 m s^−1^) and (B) the dynamic case (1 m s^−1^). The scale on the *x*-axis is indicated by the size of the icons, which represent 0.5, 1, 1.5 and 2 mm particles and 3 times 1.4 mm particles from left to right.

Furthermore, a notable phenomenon was observed in the magnitude of the drop in absorption between the static and dynamic cases: while the SoA particles outperformed the newly developed particles in the static case, their performance decreased more steeply in the moving case. In Fig. 3 it can be seen that the particle with only 1.0 mm diameter performed comparably to the SoA particles, which were 40% larger in diameter and 270% larger in volume. Consequently, especially for quantifying quickly moving flows, the novel particles excel. This effect presumably stems from the difference in how radio-opacity is distributed within these two types of particles. The SoA particles are only radio-opaque in the middle, because of their design with the small metal rod inside the particle. During fast movement, the 0.4 mm diameter rod moved 0.5 mm within one frame, as the shutter speed was set to 1/2000 s. Together with potential ghosting artefacts on the image intensifier's scintillator screen, this led to a relatively large broadening of the peak.

As in this example case, the absorption of the metal is spread over twice the distance, so the roughly halving of the SNR peak of the SoA particles seems logical. The newly developed particles, however, have a homogeneous distribution of radio-opaque material, the metal powder. Hence, they are less susceptible to motion-blurring effects than the SoA particles because of their broader absorption spot. In conclusion, the SoA particles are better in the non-moving case, but for fast motion, as usually seen in animals during XROMM, the new particles outperform the old particles and even allow the use of particles with a 29% smaller radius and 63% smaller volume, enabling tracing of flows at a higher resolution.

**Fig. 4.**
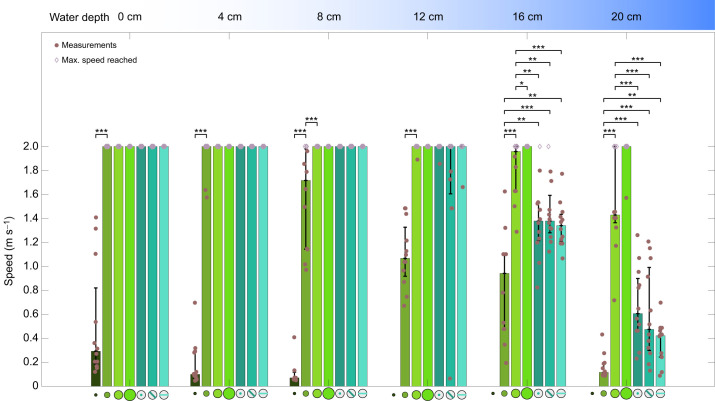
**Bar chart for traceability of the various particles on high-speed X-ray video as a function of their movement speed (vertical axis) and water depth.** Particles are in the same order as in Fig. 3. Note that the measurements (circles) had to be truncated at our maximum experimental speed of 2 m s^−1^. Maximum speed reached is indicated by diamonds. Black whiskers indicate the 25th–75th percentile. The brackets above the bars show the significance level of differences between two particles (permuted Brunner–Munzel test: *P≤0.05, **P≤0.01, ***P≤0.001).

The third and most critical parameter for evaluating particle performance is their suitability for automatic tracing. To quantify this, the following methodology was applied: if a particle required readjustment two times within five frames, the speed before the second readjustment was defined as the maximum tracing speed. For the SoA particles, it must additionally be considered that the maximum tracing speed has to be taken as the minimum of the three tested orientations, as under normal conditions the particles would rotate and continuously change orientation. Notably, the 1.0 mm particle performed almost as well as the 1.4 mm SoA particles up to a water depth of 16 cm (Fig. 4). This indicates that particles with a 33% smaller diameter can be used for the same application. The 1.5 mm particles consistently outperformed the SoA particles of comparable size. In our benchmarking, the 2.0 mm particle was always traceable at the highest speed available during the validation experiment (i.e. 2 m s^−1^). The 0.5 mm particles could be traced at low water depths and relatively low speeds, but they remained usable at depths up to 8 cm. These results demonstrate that the new particles not only match but in several cases significantly surpass SoA performance, allowing for smaller particle sizes without compromising traceability.

The developed particles provide an effective solution for flow tracing in XROMM experiments involving living animals. Our results show that similar-sized particles significantly outperform the SoA particles, allowing for a more precise description of flow patterns. Through the design of the manufacturing, the particle diameter can be adjusted to specific experimental needs, allowing us to choose an appropriate traceability by simply scaling the 3D-printed mould (available from https://www.thingiverse.com/thing:7278079). The newly designed production process also enables the creation of particles with lower density variability and yields more than twice as many particles in the same amount of time. This higher and more consistent production rate is particularly valuable, as tracing experiments typically require large particle numbers. The 1 mm particles were successfully employed in feeding trials with live ducks, where they withstood the filtration process and remained traceable even in high-speed (>>1 m s^−1^) flow conditions. This indicates that the particles were well tolerated, as the animals continued feeding normally after ingesting them. Additionally, particles that remained in the tank can be scooped out and stored for later use. All together, these results highlight the potential of the new particles to enhance X-ray particle tracing and to improve our understanding of complex flow dynamics in and around animals.

## Supplementary Material

10.1242/jexbio.252187_sup1 Supplementary information
