# Supplementary material for: A low-cost, high-performance method for the production of particles to trace water flow with high contrast in XROMM studies
Source: J Exp Biol. 2026 Jul 13;229(13):jeb252187. doi: 10.1242/jeb.252187 (PMC13405214; doi:10.1242/jeb.252187)
Supplement: Supplementary information [file jexbio-229-252187-s1.pdf]

Supplementary Materials and Methods

Recipe

This recipe will guide you step by step through the production process of the tracing particles, described in “Low-cost, high-performance method for the production of particles to trace water flow with high contrast in XROMM studies”.

Prepare all the different components as stated in Table 1. Start weighing with the solid components, as the liquid ones will start to evaporate as soon as they are put on the scale and will make the measurement more difficult. We advise using the same container, as otherwise the loss due to changing the container is unknown. In our test series, we made 5 grams of the mixture. Once the mixture is weighed, use a silicone spatula to mix it by hand. Avoid high shear forces; this means not dragging the spatula flat across the surface of the container, as this was found to increase the density of the mixture. The material is mixed for 10 minutes; make sure to mix it well so that the mixture appears visually homogeneous, meaning that there are no spots of single material left, as shown in Fig 1a. This mixture is now ready to be worked on and is best stored in a closed container. The shelf life is around 2 months, as it will dry out slowly over time and become more difficult to work with.

The material is now put into a syringe with Luer lock and an opening of 2 mm, and pressed through the syringe to create a rough cylindrical shape. Depending on the size of the spheres, only half a centimetre to a few centimetres is needed to create a roll for 10 particles. Use a scalpel to cut a piece of the extruded cylinder and then place it on a flat glass surface. It is useful to put height separators on either side so it is clear when the desired diameter of the roll is reached. Now use a second piece of glass to roll it flat. It is important not to exert too much force, meaning the roll should not be pressed into a flat shape, as this will most likely cause smearing and the roll to fall apart. If this happens, it is best to remove the mixture from the surface and clean it with ethanol, because once the mixture adheres to the glass it will also negatively affect following rolls. With gentle pressure and movement back and forth, roll the cylinder into the desired diameter; as mentioned in the manuscript, this should be roughly 87% of the intended sphere diameter. Separators can either be printed with a 3D printer or made from another material that has the correct height. If it is not possible to get a separator with the correct height, one can estimate it by putting the roll into one of the grooves of the 3D-printed mold. This gives a good estimate of the diameter of the roll; it should be visually smaller than the diameter of the groove.

Once the diameter of the roll is correct, the roll is placed perpendicular to the grooves and, with a scalpel, cut so that its length equals the width of the 10 grooves of the mold. The second part of the mold is then placed gently on top, and by moving it back and forth, the grooves of the mold will be pressed onto the roll, shaping it into spheres; after rolling with little pressure, this looks like in Fig 1d.

By continuing with increasing pressure, the grooves of the mold will “cut” the roll into spheres. If the diameter of the roll is too small, it will result in donut-shaped particles, as seen in Fig S1a; on the other hand, if the diameter is too large, it will result in smearing of the mixture, as seen in Fig S1b.

Once the particles look like in Fig 1e, they are ready to be hardened in the oven. The tested routine was to create a few hundred particles in this way, store them on the side, and bake them all together in one go. For this, the baking time stated on the package of the liquid clay was used. In the case of the Fimo that we used, it was 20 minutes at 130°C. This was done in a preheated oven on a metal tray that was also preheated to keep the temperature as stable as possible, and it was constantly monitored with an external thermometer to make sure that the temperature stayed within ±5°C of 130°C.

After baking, the particles were directly put into water and then stored for 7 days before use in the experiment. After the 7 days, the density was stable and the particles were ready to use.

A

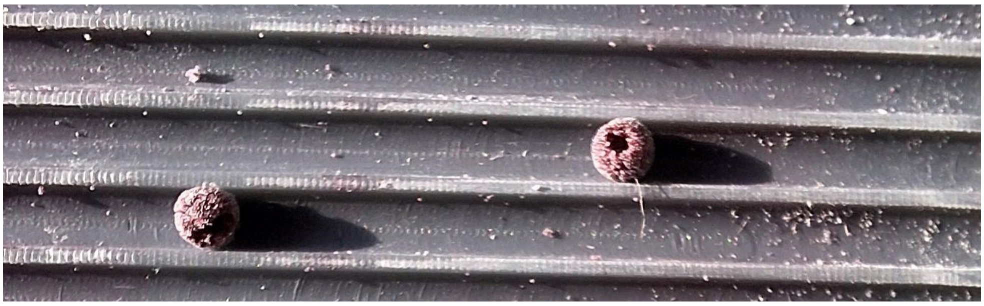

B

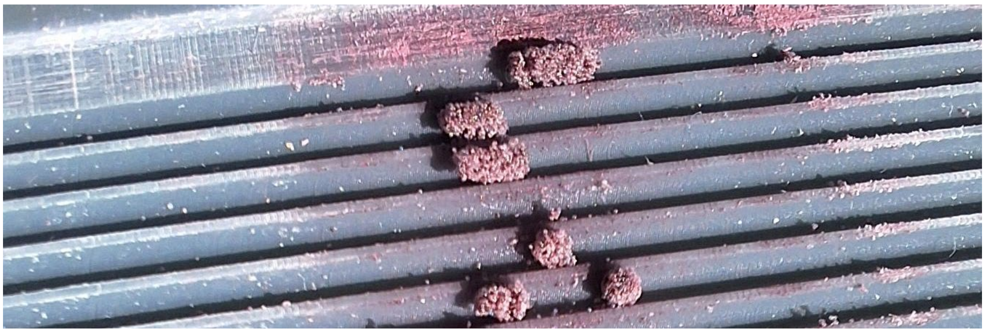

Fig S1. Failure images for the wrong diameter of the roll. In A, the diameter of the roll was too small and resulted in donut-shaped particles. In B, the diameter of the roll was too large and resulted in smearing of the mixture rather than forming spheres.

Journal of Experimental Biology • Supplementary information
